# Single institution analysis of incidence and risk factors for post-mastectomy pain syndrome

**DOI:** 10.1038/s41598-018-29946-x

**Published:** 2018-07-31

**Authors:** Lingfei Cui, Ping Fan, Chaoxue Qiu, Yong Hong

**Affiliations:** Nanxishan Hospital of Guangxi Zhuang Autonomous Region, Guangxi Zhuang Autonomous Region Second People’s Hospital, Breast surgery, Guilin, Guangxi China

## Abstract

Post Mastectomy Pain Syndrome (PMPS) is a common postoperative condition for breast cancer, but has been ignored. The aim of this study was to investigate the prevalence of PMPS and the risk factors in women submitted to surgical treatment for breast cancer. The study included 532 postoperative breast cancer patients in a hospital for five consecutive years period, of whom 473 were considered eligible and included in the study. A total of 420 people completed a questionnaire survey, which revealed that 152 (36.2%) had ever suffered from PMPS and 18 (11.8%) sought treatment. Of the patients with PMPS, 34 (22.4%) had a history of chronic pain. Patients with PMPS were younger than patients without PMPS (50.5 ± 10.4 vs 53.5 ± 11.1). Univariate analysis showed that age, history of chronic pain, tumor staging, number of lymphadenectomy were significantly different between the two groups. Multivariate analysis shows that age and history of chronic pain were independent risk factors. The incidence of PMPS in postoperative breast cancer patients can reach 36.2%, and age as well as previous history of pain are independent risk factors for PMPS. The combination of prevention and treatment may be an effective way to reduce PMPS.

## Introduction

Breast cancer is the most frequently diagnosed cancer globally and the leading cause of cancer-related death in women^1^. About 255180 people were diagnosed with breast cancer in the United States in 2017^2^. In China, breast cancer is the most common cancer among women and expected to account for 15% of all new cancers in women^3^. Mastectomy remains the main treatment for breast cancer, and nearly 37–40% of women with breast cancer receive the surgery^4^. About 20–68% of these patients experience post-mastectomy pain syndrome (PMPS), which is characterised as a dull, burning and aching sensation in the anterior chest, arm and axilla and exacerbated by movement of the shoulder girdle, continues more than three months after the surgical procedure^5^. PMPS seriously affects the physiological, psychological and quality of life of breast cancer patients^6^, and these patients are a large group and should attract our attention.

It has been decades since the first case of PMPS was reported^7^, and early studies underestimated the incidence of PMPS for a variety of reasons. Although there is no uniform standard for its incidence, most studies have indicated that its incidence is high. Still, many doctors do not think it is a common symptom and ignore its prevention and treatment. A large number of patients with PMPS who have sought outpatient consultation did not know that there are treatment methods available and never were treated for the pain. Many gave up the idea that their pain could be alleviated^8^. Although some surgeons engaged in breast cancer practice recognized the incidence and duration of PMPS, sufficient treatment had not been provided to the patients, and the treatment effects were insufficient^9^.

PMPS’s research in China is lagging and neglected. Due to the differences in race, disease prevalence, surgical techniques, and timing of surgery, it is necessary to study the prevalence and risk factors of PMPS in China. The aim of this study was to investigate the prevalence of PMPS and the risk factors in women submitted to surgical treatment for breast cancer.

## Methods

### Study Population

The experimental design was a retrospective study. We investigated all breast cancer patients who underwent mastectomy in the Guangxi Zhuang Autonomous Region Second People’s Hospital between January 2011 and December 2016. Patients who had experienced any of the following were excluded from the study: male patients, preoperative pain around the affected breast, bilateral breast cancer, with an operation history, with mental illness and unable to communicate. Patients who met the requirements were informed of the purpose of the study and informed consent was obtained from all participants. The study was approved by the ethics committee of the Guangxi Zhuang Autonomous Region Second People’s hospital. The methods involved in the study were carried out in accordance with the relevant guidelines and regulations.

### Definition of PMPS

The definition of PMPS was based on the results of previous studies and pre-experiments, which included the following three criteria: character of the pain should be typical of neuropathic pain, such as numbness, needle, burning; location of pain should be in the ipsilateral axilla, arm, shoulder or chest wall; timing of the pain lasted more than three months.

### Medical Record Collection

Relevant data was collected from medical record. Risk factors such as residence (urban, rural), marital status, occupation, education, preoperative body mass index, tumor staging, surgical procedure, lymph node dissection, chemotherapy, chemotherapy regimen, hormone therapy, were recorded.

### Study questionnaire

A self-designed questionnaire based on the Short-Form of McGill Pain Questionnaire (SF-MPQ) was used to survey the patients involved. The questionnaire determines whether postoperative pain exists or not. For patients with postoperative pain, duration of pain, pain grading, characteristic of pain and location of pain were recorded. Only those postoperative pain that meet the three criteria we have defined, as mentioned above, are considered PMPS. A body chart is attached to the questionnaire, on which participants can mark the pain position directly to avoid ambiguity. The contents of the investigation also included: aggravation or not when moving shoulder, intervention or not, relief or not after intervention, and impact or not on life.

### Study Procedures

The survey was conducted by the trained professionals who instructed participants to complete the questionnaire. Patients included in this study received a telephone interview to be informed the purpose of the study. Further study can be carried out with the consent of the patient. Then, a questionnaire with a cover letter and a stamped addressed reply envelope be sent to the patients. Those patients who failed to respond within two weeks were contacted again for reasons, if necessary, mailed the questionnaire again or inquired to complete the questionnaire on telephone. And some patients completed questionnaires at outpatient visits. Pilot studies of these procedures and of the questionnaire were undertaken prior to the main study.

### Statistical analysis

Statistical computations were performed in SPSS 24.0. The measurement data were expressed as mean ± standard deviation, the counting data were expressed as examples and percentages, and the t test and chi-square test were carried out respectively. Factors with statistical significance in univariate analysis were included in Logistic multivariate regression analysis. P < 0.05 was considered as statistically significant.

### Data availability

All data generated or analysed during this research are included in this published article.

## Results

### Patient identification and response

A total of 532 patients underwent mastectomy procedures in our hospital over a six year period, 473 patients met the requirements and were sent questionnaires. 420 questionnaires were completed, and the reasons for not completing the questionnaire included: 1. The patient died, 2. The patient could not complete the questionnaire, 3. The patient or his family did not cooperate with the questionnaire 4. The patient could not be reached. The following analysis was based on the 420 questionnaires. The vast majority of which were total mastectomy (96.4%); Excision of pectoralis major and/or pectoralis minor in 10 cases (2.4%); Four patients underwent a second operation in six years; The number of patients with PMPS was 152 (36.2%).

### Characteristics of the study population

The age ranged from 32 to 80 years old, the average age of 52.4 ± 11.0 years. The urban population was 168 and the rural population was 252, representing 40.0% and 60.0% respectively. The number of people with higher education was 190 (45.2%), and those without higher education was 230 (54.8%). 358 (85.2%) were currently married and 62 (14.8%) without spouses. 167 (39.8%) were unemployed, and the remaining 253 (60.2%) had full-time or part-time jobs. 66 (15.7%) patients had a history of chronic pain. 162 (38.6%) patients were at an early stage, while 258 (61.4%) in advanced stages. The number of lymph nodes removed ≥15 in 264 (62.9%) patients and <15 in the remaining 156 (37.1%) patients. Postoperative complications appeared in 42 (10%) patients. 377 (88.8%) received chemotherapy before and/or after surgery. 67 (16.0%) received radiotherapy prior to and/or after surgery. 272 (64.8%) took hormonotherapy before and/or after surgery. (Tables 1 and 2)

**Table 1:** Univariate analysis of counting data.

Characteristics	Patients with PMPS (n = 152)	Patients without PMPS (n = 268)	χ^2^	P value	
Domicile	Urban	68	100	2.227	0.136
	Rural	84	168		
Employment status	Employed	98	155	1.784	0.182
	Unemployed	54	113		
Marital status	Married	126	232	1.04	0.308
	Without spouse	26	36		
Education status	With higher education	66	124	0.317	0.573
	without higher education	86	144		
History of chronic pain	Yes	34	32	7.963	0.005
	No	118	236		
Tumor stage	early stage	49	113	4.043	0.045
	advanced stage	103	155		
Lymphadenectomy	≥15	106	158	4.829	0.028
	<15	46	110		
Complications	No	134	144	0.418	0.518
	Yes	18	24		
Chemotherapy	No	16	27	0.022	0.883
	Yes	136	241		
Radiotherapy	No	127	226	0.044	0.835
	Yes	25	42		
Hormonotherapy	No	49	99	0.940	0.332
	Yes	103	169

**Table 2:** Univariate analysis of measurement data.

Characteristics	Patients with PMPS (n = 152)	Patients with PMPS (n = 152)	T Value	P Value
Age	50.5 ± 10.4	53.5 ± 11.1	2.704	0.007
Height	160.3 ± 4.4	160.4 ± 4.3	0.364	0.716
weight	58.6 ± 6.8	59.0 ± 7.1	0.587	0.558
BMI	22.9 ± 2.8	23.0 ± 3.0	0.444	0.657

### Characteristic of PMPS

Axilla of surgical side was the most common part of the pain, and 66 (43.4%) patients reported the pain at this location. Chest wall pain was also more common, 49 (32.2%) patients reported pain at the location. There were 26 (17.1%) cases of ipsilateral arm pain, and 8 (5.3%) cases of pain in multiple location. Pain was characterized by numbness in 73 (48.0%) and burning in 45 (29.6%), followed by stabbing in 28 (18.4%) and Other indescribable pain in 6 (3.9%). In 47 (30.9%) cases, it was reported that pain could be aggravated by moving upper limbs, clothing friction, or tiredness. The duration of pain was quite different, from 3 months to more than a year. The pain in 26 (17.1%) patients was persistent, while the rest was intermittent. The frequency of occurrence also varied greatly, from 1 per month to more than 10 per month. 18 (11.8%) sought treatment, of which 12 took painkillers and 6 received physical therapy.

### Prevalence of PMPS

There were 152 people reported postoperative pain consistent with the definition based on the characteristics, location, and time of pain. Another 83 had postoperative pain, which did not fit the PMPS definition. In the patients with PMPS, 22.4% (34/152) had a history of chronic pain, while in the patients without PMPS, the proportion was 11.9% (32/268). Patients with PMPS (average age 50.5 ± 10.4) were younger than patients without PMPS (average age 53.5 ± 11.1). In the patients with PMPS, the proportion of patient in advanced stage was 67.8% (103/152), while 57.8% (155/268) in those without PMPS. 106 (69.7%) patients removed more than 15 lymph nodes in the patients with PMPS, and 158 (59.0%) in those without PMPS.

### Risk factors for PMPS

Univariate analysis showed that age, history of chronic pain, tumor staging, number of lymphadenectomy were significantly different between the two groups. The four factors were analyzed by Logistic step regression analysis and the difference was significant in history of chronic pain and age (P = 0.01, P = 0.03 respectively). It was indicated that the history of chronic pain and age has a great influence on the onset of PMPS, and was an independence risk factor for PMPS. (Table 3)

**Table 3:** Logistic multivariate regression analysis of risk factors for post mastectomy pain syndrome.

Characteristics	Wald	Odds Ratio	95%CI	P Value
Age	8.690	0.970	0.951–0.990	0.003
History of chronic pain	10.743	0.395	0.226–0.688	0.001
Tumor stage	11.255	0.757	0.466–1.232	0.263
Lymphadenectomy	1.132	0.765	0.166–1.254	0.287

## Discussion

The hospital in this study is a city-level third class A hospital, which is the main medical institution for mastectomy in China. At this level of hospital, the distribution of patients is similar to that of the real world and the result of the surgery is stable, therefore, the result has strong representativeness. In this study, the incidence of PMPS was 36.2% according to the definition. Patients in advanced stage, removed more than 15 lymph nodes, with younger age and with a history of chronic pain had a higher incidence of PMPS. Multivariate analysis showed that age and history of chronic pain were independent risk factors for PMPS. Numbness and burning were the most common pain characteristics in PMPS. Pain timing varied greatly in PMPS. Only a few patients chose to take painkiller, while fewer chose to go to medical institutions.

Persistent pain after mastectomy was first reported in 1978, and named post-mastectomy pain synthesis since then^7^. Although known as post-mastectomy pain syndrome, it was also often used in the study of other types of breast surgery^10^. Until now, there has been no well-established definition of post-mastectomy pain syndrome^11^. In 1986, the International Committee on pain Research defined PMPS as persistent pain in the forechest, axilla, and / or medial upper arm, which occurs soon after mastectomy or lumpectomy^12^. Many studies had added more than three months to the IASP definition of PMPS^5,13,14^. In fact, IASP did not define a specific time for PMPS, only that lasting more than 3 months ccould be defined as chronic pain^12^. Because there is no accepted and uniform definition, PMPS had been defined differently in different studies. Smith WC *et al*. defined PMPS through three factors in a retrospective study on PMPS risk factors: Pain is characterized by typical neuralgia with numbness, acupuncture, burning, etc; The location of the pain is the axillary, arm, shoulder, or chest wall of the operation; The pain last more than three months^13^. In an epidemiological study on PMPS, Vilholm OJ *et al*. identified PMPS as a pain located at the surgical side of the upper limb, lasting at least four days a week, and the pain intensity was greater than or equal to grade 3 on the 0–10 pain scale^5^. Couceiro TC *et al*. defined PMPS as pain in the anterior chest, axilla, shoulder and upper anterior wall after mastectomy or partial mastectomy^14^. Waltho D *et al*. Defined PMPS as neurogenic pain occurring after breast surgery, located in the ipsilateral breast or chest wall, armpit, upper arm, and lasting at least 6 months. In addition, the pain lasts at least 50% of the time, moving the shoulder joint can make the pain worse^11^.

However, according to previous studies, the incidence of PMPS varies from 20% to 65%. The study from Smith WC *et al*. included a total of 511 patients, of whom 408 completed a questionnaire survey, the number of PMPS patients was 175 and the incidence rate was 43%^13^. The study by Vilholm OJ *et al*. included 258 patients, 219 of whom completed a questionnaire survey, according to their definition, the incidence rate was 23.9%^5^. The study by Couceiro TC *et al*. included 250 patients, of whom 111 had PMPS, with an incidence rate of 44.4%^14^. The difference was caused by a variety of reasons, such as different patient choices, different tools and methods for measuring pain levels, different follow-up periods and methods. The definition in our study was based on three aspects: the characteristics, location and timing of pain, which is a generally accepted method of evaluating PMPS. Each item had a clear description, avoiding the subjective influence of the investigator. In addition, the difference of sample size and distribution is also an important factor leading to the different incidence. This study included all breast cancer patients in our hospital for six years period, and reflected the true distribution of breast cancer as much as possible.

A large number of studies have suggested that age is a risk factor for PMPS^13–18^, and our study had got the same results. A possible explanation for this is that the tumors in young patients are more aggressive and lead to more adverse symptoms. At the same time, doctors have a stronger desire to apply more invasive surgical and adjuvant treatments to improve prognosis^5,17^. Another possible reason is that anxiety about poorer prognosis leads to the development of PMPS. Anxiety is known to be closely associated with pain in different clinical situations^19^. And there is no lack of research to the contrary. In Shahbazi R’s study^20^, there was no correlation between age and PMPS. The author thought that cancer type, surgery, and surgical timing were standardized among different age groups, as a result, some distractions were removed, leading to age independent of PMPS. However, the sample size of the study was small and therefore less persuasive.

We also found that patients with a history of chronic pain had a higher risk of PMPS, which can be explained by central sensitization. Central sensitization means that long-term pain stimulation may promote the pathological remodeling of the central nervous system, which is considered to be an activity-dependent functional plasticity. It is the result of phosphorylation of key membrane receptors and channels by cascade activation of different intracellular kinases, resulting in increased synaptic efficacy^21^. The amplitude of patients’ response to pain increased and the threshold of response decreased, which may be the reason for the increased incidence of PMPS in patients with a history of chronic pain. There have been studies on the relationship between chronic pain and PMPS. In a study by Couceiro TC, it was found that the patients with a prior history of headache had a higher risk of developing PMPS^14^.

Axillary lymph node dissection is necessary in breast cancer surgery, irrespective of which type of operation is elected. Axillary lymph node dissection is associated with PMPS, which has been found in this and previous studies^22,23^. More lymph node excision indicates high incidence of PMPs. A systematic review and meta-analysis on predictors of persistent pain after breast cancer surgery showed that women who underwent axillary lymph node dissection experienced a 21% increase in the absolute risk of chronic postoperative pain^24^. Damage to the intercostobrachial nerve (ICBN) is believed to be implicated in both PMPS and permanent loss of sensory function in the region supplied^25,26^. The ICBN is a nerve classically originating from the lateral cutaneous branch of the second intercostal nerve and the most commonly injured nerve during mastectomy^27^. However, omission of axillary staging is not an appropriate approach to modifying pain risk, for the risks of omitting axillary nodal sampling include increasing the number of patients who are understaged and undertreated and who experience reduced survival^28^. Instead, modification of surgical procedures related to axillary dissection constitutes a promising stand-alone target for risk reduction. Preservation of the ICBN is an effective way to reduce PMPS^29^ and should be factored into operative plans. Differences in the neural division result in the fact that this method is not always effective, sometimes, nerves which are successfully identified and protected can still present with postoperative complications. It was noted that six months following the procedure, 20% of patients who had successful ICBN preservation still experienced numbness and paresthesia. Sentinel lymph node biopsy, rather than standard axillary treatment, may reduce the risk of chronic pain after breast cancer surgery with no increase rates of axillary relapse^30^ and be recommended for patients with early-stage breast cancer, followed by dissection only if the biopsy result is positive^31^.

Facing such a high incidence, PMPS should not continue to be ignored by patients and doctors. Preservation of the intercostal brachial nerve could decrease chronic pain and the use of sentinel lymph node biopsy could reduce the need for axillary dissection and thereby intercostal nerve damage. COX inhibitors, which are considered not suitable for preventing neuropathic pain, can only alleviate the postoperative inflammatory pain^9^. However, they might also work for neuropathic pain, because central sensitisation can be prevented by aggressive early pain relief^32^. In addition, epidural or regional blockade of the afferent input to the central nervous system can achieve similar effects, but unlike COX inhibitors, has a remarkable effect on reducing the incidence of PMPS in one year after operation^33,34^. Antidepressants and antiepileptic drugs have also been used in the treatment of PMPS and promising results have been obtained in the reduction of PMPS with perioperative administration of venlafaxine^35^ or gabapentin^36^. Instead of for inflammatory pain, these treatments are for neuropathic pain. Other studies found venlafaxine or gabapentin can only improved acute postoperative pain and further data were needed on reducing chronic postoperative pain^37,38^. Some physiotherapy, including acupuncture^39^, laser therapy^40^, autologous fat grafting^41^, etc., have also been used in the treatment of PMPS and achieved some results. Importantly, future studies of the effect of preventive analgesia on the risk of PMPS should focus on the completeness and appropriateness of the intervention rather than on the timing of analgesia.

### Study limitations

The limitations of this study are its consideration of patients only at a single center, a retrospective study, nonrandomized design, and a relatively small sample size. Further high-quality, prospective, multicenter studies were necessary to fully elucidate the incidence.

## Conclusion

The incidence of PMPS in postoperative breast cancer patients can reach 36%, which should get doctors’ attention. Age and previous history of pain are independent risk factors for PMPS and the patients with younger age or a history of chronic pain women may be at a higher risk of developing PMPS. The number of PMPS patients treated is limited, and surgeons should give patients undergoing breast cancer surgery more information about PMPS and actively provide effective treatment.
